# Landscape of esophageal cancer in Northern Kenya: experience from Garissa Regional Cancer Center

**DOI:** 10.3332/ecancer.2024.1694

**Published:** 2024-04-11

**Authors:** Omar Abdihamid, Houda Abdourahman, Abdulsadiq Ibrahim, Thinwa Kareu, Abdullahi Hadi, Abeid Omar, Miriam Mutebi

**Affiliations:** 1 Garissa Regional Cancer Center, Garissa County Referral Hospital, PO Box 29-70100, Garissa, Kenya; 2 Department of Pathology, Hopital De Balbala Cheiko, Balbala, PO Box 669, Republic of Djibouti; 3 Department of Gastroenterology, University of Pretoria, PO Box Prinshof 349-Jr, Pretoria 0002, South Africa; 4 Kenyatta University Teaching Research and Referral Hospital, PO Box 7674 – 00100, GPO Nairobi, Kenya; 5 Department of Surgery, Aga Khan University, PO Box 30270-00100, Nairobi, Kenya; † Shared first authors

**Keywords:** esophageal cancer, incidence, mortality, Garissa, Kenya

## Abstract

**Introduction:**

Esophageal cancer (EC) is the ninth most common cancer and the sixth leading cause of cancer deaths worldwide. More than 80% of cases and deaths from EC occur within developing countries. In Kenya, cancer is the second leading cause of non-communicable disease deaths, and the trend of cancer deaths is projected to increase as per the 2020 GLOBOCAN report showing 42,116 new cases annually with a mortality of 27,092 cases. EC is the leading cancer in men and the third most common in women in Kenya. The Garissa Regional Cancer Center (GRCC) is one of the three regional cancer centres in Kenya. Despite the rising EC incidence in the region, there is limited data about the clinicopathological features and treatment outcomes of EC, therefore, this is the first study to look at the landscape of EC in the northern Kenya region.

**Methods:**

This was a retrospective study involving patients’ file review of confirmed EC cases diagnosed or treated at the GRCC from 2019 to 2023. Data collected from each patient’s chart included age, sex, risk factors, family history of EC, histological type, stage at diagnosis, treatment type and survival outcomes. For patients who were no longer in contact with the staff through clinic visits, the patients or their next of kin were contacted through phone calls for patients’ survival status. Data were collected and stored using the STATA software.

**Results:**

Over the study period, 124 esophageal cases were identified, 64 (51.4%) were males and 60 (48.4%) were females with a mean age of 57.56 years. In terms of risk factors, hot beverage consumption was the highest (47 cases, 37.9%), followed by history of peptic ulcer disease (27 cases, 21.8%), smoking (8.9%) and gastresophageal reflux disease (2 cases, 1.6%). Stage of diagnosis at presentation was stage 1 (1 case, 0.8%), stage 2 (22 cases, 17.8%), stage 3 (25 cases, 20.2%), stage 4 (50 cases, 40.3%), not staged (26 cases, 21%). The majority had squamous cell carcinoma (SCC) (105 cases, 84.7%), followed by adenocarcinoma (5 cases, 4%), anaplastic (5 cases, 4%), SCC+ adenocarcinoma (1 case, 0.8%), unknown histology (8 cases, 3.2%). Nearly all patients had triple assessment (Endoscopy, histology and staging scans) accounting for 92 cases (74.2%), 24 cases (20%) had endoscopy+ histology only, and 8 cases (3.2%) had only imaging scans. In terms of family history of EC, 20 cases (16.1%) had a family history of EC.

Most of the patients were of ethnic Kenyan-Somali background (108 cases, Kenyan Somali, 87.1%) and majority were from Garissa County 96 cases (77.4%), 12 cases (9.7%) Wajir County, 12 cases (9.7%) from Tana River County and 4 cases (3.2%) from other counties. Many patients lacked health insurance (27 cases, 25.8%), while the majority paid out of pocket (92 cases,74.1%). Only 21% (26 cases) received chemotherapy alone, 5 cases (4%) got radiotherapy alone, 12.9% (16 cases) got chemoradiotherapy and a significant number of patients (77 cases, 62.1%) did not receive hospital-based cancer treatment.

**Conclusion:**

This study is the first esophageal study at the GRCC and in northern Kenya in general. Our study confirmed the clinicopathological features of one of the most common cancers in Kenya and more so among Kenyan-Somalis.

The study also validates the predominance of histological subtypes of esophageal SCC with the late presentation, short survival and significant loss of follow-up. We recommend future EC studies employing a large prospective design with a large sample size to determine the impact of the new GRCC on the outcomes of EC patients and the local community.

## Background

Esophageal cancer (EC) is the ninth most common cancer and the sixth leading cause of cancer deaths worldwide [1]. More than 80% of cases and deaths from EC occur within developing countries [2, 3]. The incidence of EC varies globally [4], with a higher incidence in areas such as Eastern Asia, South Central Asia, South Africa and Eastern Africa [3, 5]. Notably, EC continues to receive great attention due to its increasing incidence throughout the eastern Africa corridor, extending from Ethiopia to South Africa, heralding increasing number of collaborative and multicentre studies on the understanding the aetiologies of high EC incidences and the formation of working groups and consortiums such as African Esophageal Cancer Consortium [6] and the esophageal squamous cell carcinoma (ESCC) African PrEvention research [7]. This collaborative research has mainly employed case-control studies to investigate risk factors for ESCC such as hot beverages, oral hygiene and dental fluorosis, and alcohol [6–10].

In terms of diagnosis of EC, triple modalities such as endoscopic biopsy, histology confirmation and staging scans are the standard of care [11]. Emerging novel approaches to screening that may be cost-effective in EC endemic regions have been explored such as the use of Lugol’s iodine staining using endoscopy to detect precancerous lesions in the esophageal mucosa by Mwachiro *et al* [12] and the use of swallowable Cytosponge [13], a form of minimally invasive esophageal cytology sampling technique to improve early detection for this debilitating disease that presents at locally advanced stage in many EC hotspots in Africa [14–18].

Overall the standard diagnostic and treatment modality remains the use of multi-disciplinary evaluation comprising oncologists, surgeons, nutritionists and palliative care specialists among others [15]. The currently available treatment strategies for EC include surgery, chemotherapy, radiation therapy, molecular targeted therapy and immunotherapy therapy [16]. However, the prognosis remains poor, and the overall 5-year survival rate of less than 20% [17]. Ongoing prospective studies will shape guidelines-based management strategies for EC in the Eastern Africa region which could potentially be a benchmark for other low-and-middle-income countries plagued with the high incidence of ESCC [18]. Due to late-stage presentation, early initiation of nutrition intervention such as placement of a feeding tube and palliative care services including optimal pain control is vital in improving the overall survival and quality of life in EC patients [19–21].

In Kenya, cancer is the second leading cause of non-communicable disease deaths, and the trend of cancer deaths is projected to increase as per the 2020 GLOBOCAN report showing 42,116 new cases annually with a mortality of 27,092 cases [22]. The same GLOBOCAN data shows prostate and esophagus to be the leading cancers in men in Kenya, while breast, cervix and esophagus are the leading cancers in women, making EC the second most common cancer in males and the third most common in women [23].

There has been an upsurge of ESCC in Kenya over the years and is the most diagnosed cancer in both males and females and the leading cancer-related mortality in Kenya [6, 24–26]. Published studies regarding the epidemiological factors that may or may not drive the high incidence of ESCC in Kenya include poor diet and nutritional insufficiencies with high intake of red meat, use of alcohol and tobacco, environmental carcinogenic exposure, intake of hot beverages and genetic susceptibility citing a remarkable positive close family history of ECs [8, 27–30]. A study on geophagia, which is the intentional practice of consuming soil, especially during pregnancy is common along the African EC corridor, and its association with the development of EC has been investigated but did not appear to have a large effect on the overall risk of developing ESCC [31]. Similarly, a study from western Kenya by Menya *et al* [8] on the role of smoking and alcohol use especially traditional brews such as *busaa* and *chang’aa* showed a population attributable fraction for >2 drinks per day of 48%, contributing to half of the ESCC burden in Western Kenya. However, this concordance of alcohol intake with high incidences of ESCC is not seen among patients in northern Kenya where alcohol intake is almost non-contributory to ESCC incidence due to religious and cultural practices.

Challenges in cancer care in Kenya are multifold; patient education and poor health-seeking behaviour often contribute to late diagnosis, lack of awareness, poor uptake in cancer screening, diagnosis-related stigma, taboos, cultural barriers to seeking treatments, exorbitant cancer treatment costs, few oncology professionals and cancer care inequities remain main drivers of poor patient outcomes. The cost of cancer care creates a severe financial burden, catastrophic spending and assets liquidation, but the existing inequity in cancer care costs lives [32–34].

The Garissa Regional Cancer Center (GRCC) is affiliated with the Garissa County Referral Hospital in northern Kenya and was officially launched in February 2023, even though oncology services including chemotherapy and radiotherapy were offered from March 2022 [35]. Despite the availability of radiation and chemotherapy services at the GRCC, and rising ESCC incidence in Kenya, there is limited data about the clinicopathological features and treatment outcomes for this tumour type in this region. Therefore, this is the first study to look at the clinical pathological features of ESCC in the northern Kenya region.

## Methods

This was a retrospective study involving patient files review of confirmed EC patients diagnosed or treated at the GRCC, a regional referral cancer centre from 2019 to 2023. Data collected on each patient’s chart included age, sex, risk factors, family history of EC, histological type, stage at diagnosis, treatment type and survival outcomes. For patients who were no longer in contact with the staff through clinic visits, the patients or their next of kin were contacted through phone calls for patients’ survival status. Data were collected and stored using the STATA software. As this was a descriptive retrospective chart review, ethics and review boards were exempted by the study institution.

## Results

Over the study period, 124 esophageal cases were identified, 64 (51.4%) were males and 60 (48.4%) were females with a mean age of 57.56 years, citing nearly the same incidence of EC in terms of gender. In terms of risk factors, hot beverage consumption such as drinking hot tea was the highest (47 cases, 37.9%), followed by history of peptic ulcer disease (27 cases, 21.8%), smoking (8.9%), gastresophageal reflux disease (2 cases, 1.6%), unknown risk factors (7 cases, 5.6%) and 24.2% (30 cases) of the patients were lost to follow up with no identifiable risk factors. Alcohol intake as a risk factor for EC was captured in the patient files, in which all patients reported no history of alcohol intake, understandably due to religious and cultural practices.

Stage of diagnosis at presentation was stage 1 (1 case, 0.8%), stage 2 (22 cases, 17.8%), stage 3 (25 cases, 20.2%), stage 4 (50 cases, 40.3%), not staged (26 cases, 21%). The majority had squamous cell carcinoma (SCC) (105 cases, 84.7%), followed by adenocarcinoma (5 cases, 4%), anaplastic (5 cases, 4%), SCC+ adenocarcinoma (1 case, 0.8%), unknown histology (8 cases, 3.2%). In terms of diagnostic modality, nearly all patients especially following the launch of the new GRCC had triple assessment (Endoscopy, histology and staging scans) accounting for 92 cases (74.2%), 24 cases (20%) had endoscopy+ histology only, and 8 cases (3.2%) had only imaging scans. In terms of family history of EC, 20 cases (16.1%) had a family history of EC and 59 cases (47.6%) were lost to follow-up.

Given the homogenous ethnic demographic of northern Kenya, the majority of the patients were of ethnic Kenyan-Somali background (108 cases, Kenyan Somali, 87.1%), other tribes (7 cases, 5.6%) and lost to follow-up (9 cases, 7.3%). While most of the patients are from Garissa County, GRCC serves a huge catchment area of neighbouring counties, therefore, 96 cases (77.4%) were from Garissa County, 12 cases (9.7%) were from Wajir County, 12 cases (9.7%) from Tana River County and 4 cases (3.2%) from other counties.

Despite huge financial toxicity related to cancer costs in Kenya, many patients at the GRCC paid out of pocket for their treatment (92 cases,74.1%) while only (27 cases, 25.8%) paid through the National Hospital Insurance Fund (NHIF). Low uptake of the NHIF program is mainly due to a lack of education or awareness. There is a general lack of awareness of the existence and the potential benefits of the NHIF program among the public in northern Kenya. However, at the GRCC we are prospectively registering all new cancer patients for the NHIF program in order to improve awareness and uptake of all health insurance programs. Furthermore, fundraising from immediate family members, well-wishers and asset liquidation are common coping financial mechanisms (Table 1).

In terms of the treatment modalities and outcomes (Table 2), 21% (26 cases) received chemotherapy alone, 5 cases (4%) got radiotherapy alone, 12.9% (16 cases) got chemotherapy plus radiotherapy and a significant number of patients (77 cases, 62.1%) did not receive hospital-based cancer treatment. Of note, there were not any EC patients who underwent esophagectomy even after achieving pathological complete response following concurrent chemoradiation mainly due to a lack of or few qualified surgeons to perform such procedures at the GRCC or at other high-volume centres.

**Figure 1.**
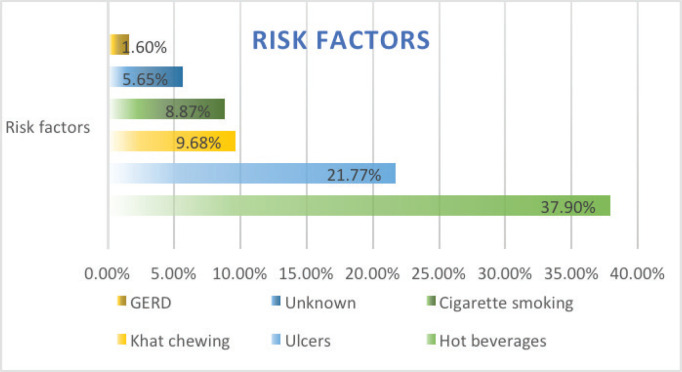
Risk factors of EC from patients’ files.

**Figure 2.**
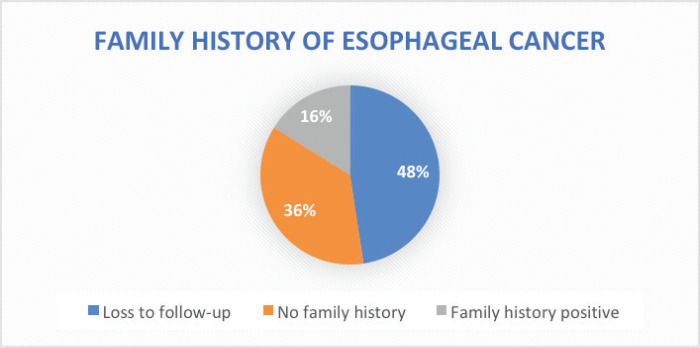
Family history of EC.

**Figure 3.**
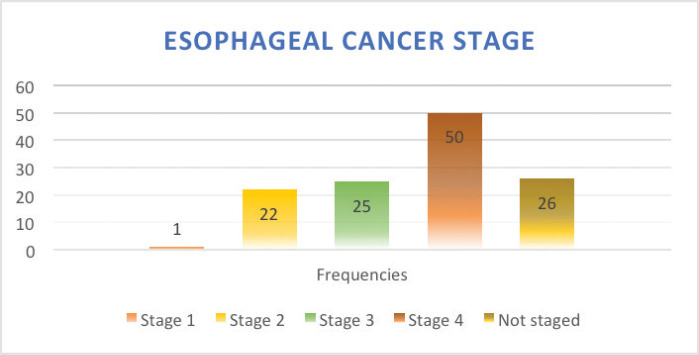
EC stage at presentation.

**Figure 4.**
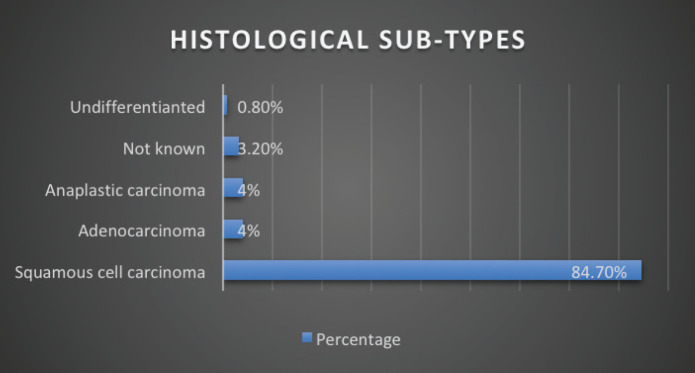
Histological subtype of EC.

**Figure 5.**
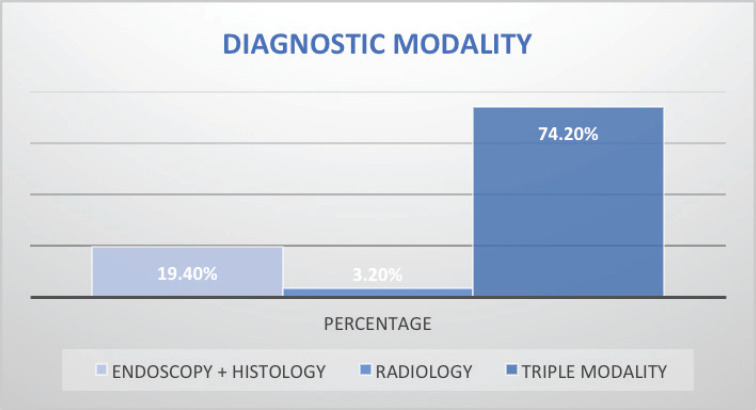
EC staging modality at diagnosis. Triple modality (Endoscopy, histology and CT scans).

**Figure 6.**
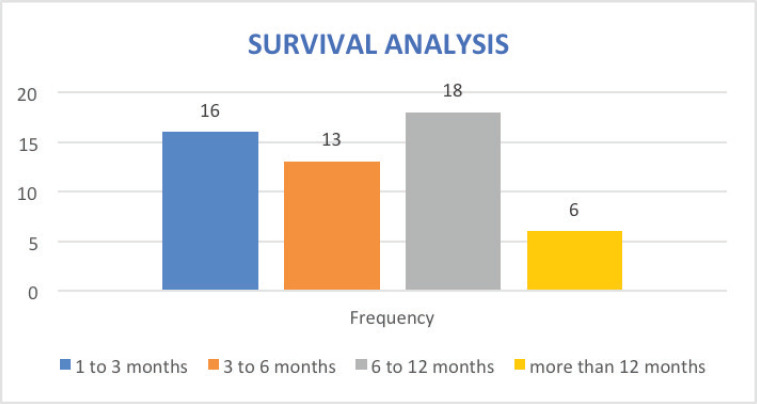
Overall survival analysis of EC patients in months.

Owing to the advanced disease stage presentation, most patients present with cancer-related cachexia, and therefore, the rate of gastrostomy insertion for nutritional support among the EC patients was 23.4% (29 cases), while the stenting rate for relieving dysphagia was 2.4% (3 cases). Survival status was confirmed through phone calls to patients or their next of kin. 55 cases (44.4%) were lost to follow-up, 41.9% (52 cases) were reported to have died, and 17 patients (13.7%) were alive at the time of the study analysis. Overall, the 1–3 months survival rate was 16%, 3–6 months was 13%, 6–12 months survival was 18% and >12 months survival was 6%. Figures 1–6 show risk factors, family history of cancer, staging at diagnosis, histological sub-type, diagnostic modality and median survival in months, respectively.

## Discussion

This is the first study that reviewed the clinicopathological patterns and presentations of EC patients and treatment outcomes at the GRCC, which is affiliated with the Garissa County Referral Hospital, Kenya. GRCC is the first cancer centre in the northern Kenya region in 60 years [36], heralding a huge milestone in the decentralisation of cancer in Kenya. The study finding also provides the first EC data specifically from the northern Kenya region that has a disproportionately high ESCC incidence [37], therefore, the findings are relevant and add to the much-needed data on the outcomes of EC in Kenya. Given, the lack of previous studies for comparison, and the homogenous Kenya Somali community in this region, this data is largely representative of both the ethnographic and epidemiological patterns of EC patients as well as their clinical outcomes.

Despite, the unique male predominance in EC in Africa [38, 39], our study showed a similar incidence of ESCC between males and females even in the absence of cigarette smoking and no alcohol use among both sexes. In our cohort, the majority of the ESCC patients were ethnic Kenyan Somalis (87%) from Garissa County (77%), followed by Wajir and Tana River counties at 9.8% and 10%, respectively, which correlates with the homogenous settlement of the ethnic Kenyan-Somali community in the northern Kenya region. This is similar to data from Somalia showing EC as the most common cancer with similar male and female EC incidence and late-stage disease presentation [40].

The highest identifiable risk factor for ESCC in our study was a history of hot beverage intake which accounted for 58%, followed by a history of peptic ulcer disease at 33%. However, a case-control study in Kenya on the association between hot beverages and ESCC concluded a lack of true association [7]. Despite recording a history of peptic ulcer disease as a documented risk factor for EC in our study cohort at 33%, the association of peptic ulcer disease with ESCC is rare according to the literature [41], and cannot confer a positive association. Therefore, a case-control study is urgently needed to ascertain the aetiology of the high incidence of ESCC in the northern Kenya region. A family history of ECs as a risk factor for EC was identified in 16% of patients. However, in the present study, we solely depended on ascertaining the status of positive family history for EC based on qualitative means (phone call) with the registered next of kin. This strategy is clearly less reliable and can be affected by several factors such as recall bias, literacy level and certainty of EC diagnosis by the family members. In the future, emerging EC strategies such as opportunistic endoscopy screening for accompanying family members during the diagnosis of EC of patients [42] and the use of risk-stratified endoscopy screening for precancerous lesions can be explored to improve early detection in patients with positive family history.

Our data demonstrates ESCC as the most common histology representing over >80% of all cases, which is in keeping with other studies from East African countries including Kenya, Tanzania, Uganda [43–46] and South African countries [47] as well as incidences from China and Iran [48, 49]. More than 50% of the EC patients were in stage IV at presentation, data that is consistent with studies from regions with high prevalence of ESCC such as Ethiopia [50], Zambia, China [49] and India [51]. A significant 20% of the patients were not staged, owing to the limited diagnostic oncological services available before the launch of the new GRCC.

Accurate staging is critical in EC. Only 74% of our cohort had a conventional triple modality diagnosis (endoscopy, histology and staging scans), while others had staging scans alone or with histology alone. Furthermore, 21% of the patients in our study had no mode of cancer staging hence their stages were unknown, possibly due lack of cancer awareness and low literacy levels. Our findings are similar to the barriers to cancer diagnosis and treatment in other parts of Kenya. A study by Makau-Barasa *et al* [34] found the high cost of testing and treatment, low level of knowledge about cancer among the population and clinicians, poor health-seeking behaviours among the population, long distances to access diagnostic and treatment services, and few comprehensive cancer centres to be key barriers to improving early access to cancer diagnosis and treatment in Kenya [34]. However, progress has been made in recent years to improve access to cancer care, especially following the launch of three regional cancer centres and the launch of national action strategic plans for 2020–2030 [33].

The cost of cancer is a big determinant of cancer treatment compliance, and only 25.8% of our cohort had a form of health insurance, while 74% paid out of pocket for treatment, leading to catastrophic spending and financial toxicities. While cash payers were high in our cohort, family fundraisers, loans, patients’ assets liquidation and sponsorships are common financial coping mechanisms among cancer patients in Kenya. The low uptake of health insurance is mainly due to a lack of patient education, low health-seeking behaviour and a lack of incentives to enroll in such programs for patients in rural settings in Kenya. There is also a general lack of awareness of the existence, and the potential benefits of the NHIF program among the public in northern Kenya. However, the institution policy at the GRCC to prospectively register all new cancer patients for the NHIF program is currently implemented and is gradually improving awareness and uptake of the insurance programs.

Owing to the unavailability of cancer treatment services before the launch of GRCC, only 21% (26 cases) had palliative chemotherapy, 13% had chemoradiotherapy and 62% had no form of documented treatment and were lost to follow-up. Moreover, due to a lack of experienced surgical oncologists, esophagectomy is not routinely done even for patients who achieve a complete pathological response following neoadjuvant chemoradiotherapy. The low esophagectomy rate also highlights the broken linkages for referral for such patients to high-volume centres with expertise in doing esophagectomies. Due to late-stage presentation and resultant severe cachexia, 23.4% (29 cases) had a gastrostomy tube insertion for nutritional support before and during treatment and 2.4% (3 cases) had esophageal stenting done.

Survival analysis shows a significant number of our patients lost to follow-up (44%, 55 cases), indicating a poor follow-up pathway and dismal survivorship care. 42% (52 cases) were reported to have died at the time of study analysis, and only 14% (17 cases) were alive. While these data paint a dismal survival outcome, there is an overlap of patients’ data prior to the launch of the new GRCC which provides conventional cancer care and may have affected the final analysis. Also, since most patients were diagnosed at an advanced stage of cancer, the overall follow-up duration for median survival was too short, leading to the estimation of time-specific survival rates being less precise. Importantly, EC carries a high mortality in sub-Sahara Africa [19] with a study from Malawi showing a median time to death of 106 days (95% CI, 92 to 127) from diagnosis. Equally the 1, 2 and 3-year survival rates were 11% (95% CI, 8 to 15), 3% (95% CI, 1 to 6) and 0.9% (95% CI, 0.8 to 4), respectively [52]. We intend to carry out subsequent large sample-size studies that will reveal the survival outcomes of EC patients seen at the GRCC after receiving the currently available standard of care.

## Strengths and limitations

The current study is the first in the northern Kenya region to highlight the general burden of EC among patients seen at the GRCC. The study revealed barriers and opportunities to explore across the continuum of care of EC patients. The study data will influence policy formulations prospectively at both levels of government in designing programs that will improve early access to cancer treatment. The study limitation is mainly a single-centre experience and the retrospective nature that draws recall bias and underrepresentation of patients lost to follow-up or those who sought care elsewhere, thus underestimating their clinical and treatment outcomes. Similarly, the overlap of patients’ data who did not get standard of care and those treated at the new GRCC, may dilute the true patterns of EC outcomes.

## Conclusion

This study is the first esophageal study at the GRCC and in northern Kenya in general. Our study confirmed the clinicopathological features of one of the most common cancers in Kenya and more so among Kenyan-Somalis. The study also validates the predominance of histological subtypes of ESCC with the late presentation, short survival and significant loss of follow-up. Our data demonstrates the need for the county government of Garissa in collaboration with the National Cancer Control Program to prioritise EC on the national health agenda, including promoting preventative strategies, cost-effective early screening, early detection and timely treatment, and designing interventions to improve treatment adherence such as using appointment reminders via mobile phones, setting up an up-to-date cancer registry, and enhancing referral pathways.

We recommend future EC studies employing a large prospective design with a large sample size to determine the impact of the new GRCC on the outcomes of EC patients and the local community. Also, given the absence of well-established EC risk factors such as alcohol consumption and cigarette habits among this community, the geographical location, and the ethnic background of homogenous Kenyan Somalis in the region, a culturally based and individualised case-control studies that incorporate local context and lifestyle practices as well as environmental studies is urgently needed to elucidate the high incidences of ESCC in this region.

## Conflicts of interest

The authors declare that they have no conflict of interest.

## Funding

None to be declared.

## Figures and Tables

**Table 1.** Social demographics and clinicopathological characteristics of EC patients at the GRCC.

Characteristics	Numbers	Percentages (%)
Male	64	51.6
Female	60	48.4
Mean age: 57.56		
Risk factors		
Hot beverages	47	37.9
Gastroesophageal disease	2	1.6
Smoking	11	8.9
Peptic ulcer disease	27	21.8
Unknown	7	5.6
Lost to follow up	30	24.2
Stage of diagnosis		
Stage 1	1	0.8
Stage 2	22	17.8
Stage 3	25	20.2
Stage 4	50	40.3
Not staged	26	21.0
Histological subtype		
SCC	105	84.7
Adenocarcinoma	5	4.0
Adenocarcinoma + SCC	1	0.8
Anaplastic	5	4.0
Unknown histology	8	6.4
Diagnostic modality		
Radiology alone	8	6.4
Endoscopy + Histology	24	19.3
Endoscopy+ Histology+ Radiology	92	74.2
Ethnicity		
Kenyan Somali	108	87
Others	7	5.6
Lost to follow up	9	7.2
Residence		
Garissa county	96	77.4
Wajir county	12	9.7
Tana River county	12	9.7
Others	4	3.2
Family history of ECs		
Yes	20	16.1
No	45	36.3
Lost to follow up	59	47.6
Payment method		
NHIF	32	25.8
Cash	92	74.2

**Table 2.** Treatment types, supportive care and survival outcomes of EC patients.

Treatment outcomes	Number	Proportion (%)
Feeding gastrostomy tube	29	23.4
Esophageal stenting	3	2.4
Treatment modality		
Chemotherapy alone	26	21
Radiotherapy alone	5	4.0
Chemotherapy + Radiotherapy	16	12.9
Surgery (Esophagectomy)	None	0
No treatment	77	62.1
Survival status at the time of the study period		
Alive	17	13.7
Dead	52	41.9
Lost to follow up	55	44.4
Survival analysis in months		
1–3 months	11	16
3–6 months	9	13
6–12 months	12	18
>12 months	4	6
